# How Insect Flight Steering Muscles Work

**DOI:** 10.1371/journal.pbio.1001822

**Published:** 2014-03-25

**Authors:** Anders Hedenström

**Affiliations:** Department of Biology, Lund University, Lund, Sweden

## Abstract

Insights into how exactly a fly powers and controls flight have been hindered by the need to unpick the dynamic complexity of the muscles involved. The wingbeats of insects are driven by two antagonistic groups of power muscles and the force is funneled to the wing via a very complex hinge mechanism. The hinge consists of several hardened and articulated cuticle elements called sclerites. This articulation is controlled by a great number of small steering muscles, whose function has been studied by means of kinematics and muscle activity. The details and partly novel function of some of these steering muscles and their tendons have now been revealed in research published in this issue of *PLOS Biology*. The new study from Graham Taylor and colleagues applies time-resolved X-ray microtomography to obtain a three-dimensional view of the blowfly wingbeat. Asymmetric power output is achieved by differential wingbeat amplitude on the left and right wing, which is mediated by muscular control of the hinge elements to mechanically block the wing stroke and by absorption of work by steering muscles on one of the sides. This new approach permits visualization of the motion of the thorax, wing muscles, and the hinge mechanism. This very promising line of work will help to reveal the complete picture of the flight motor of a fly. It also holds great potential for novel bio-inspired designs of fly-like micro air vehicles.

## Abstract 2

New imaging research in the blowfly reveals the dynamic movement of the steering muscles and the complex hinge mechanism in flies that enable astonishing flight maneuverability.

The ability for powered flight has evolved four times in the animal kingdom and, thanks to their ability to fly, insects have diversified and moved into new regions and habitats with enormous success [1]. Powered flight requires an integrated system consisting of wings to generate aerodynamic force, muscles to move the wings, and a control system to modulate power output from the muscles. Insects are bewilderingly diverse with respect to flight morphology and behaviors, which in turn provides a real challenge to researchers wishing to understand how insects fly. In particular, the impressive flight maneuvers in flies, such as blowflies and fruit flies, have inspired scientists for many years [2]. The ability of a fly to accelerate, make tight turns, rolls, and loops that allow the creature to land upside down on a ceiling is unparalleled in any other organisms, as well as any manmade aircraft. Everybody knows how difficult it is to swat a fly with bare hands—the fly's capacity for rapid take-off and accurate movement away from a perceived approaching threat is exquisite [3].

The flight muscles of many insects, including flies, bees, and mosquitoes, are divided into a few large power muscles that simply contract cyclically to generate sheer power output and a greater number of smaller steering muscles that control the force transmission from the power muscles to the wing [4]–[6]. The power muscles of a fly consist of two sets of antagonistic muscles attached to the inside of the thorax (exoskeleton) (Figure 1). In many insects, including flies, these muscles are asynchronous, which means their contractions are uncoupled to the firing rate of the associated motor neuron [6],[7], i.e., the muscles continue to contract as long as the nerve tickles them. Another characteristic feature of the power muscles is that they are stretch-activated and contract as a response to being lengthened. Both sets of power muscles deform the thorax when contracted such that when the dorso-ventral muscles contract, the thorax is squeezed together dorso-ventrally while expanding longitudinally, and vice versa when the dorsal-longitudinal muscles contract as a response to prior lengthening. The result is an alternate contraction and lengthening of these perpendicular muscle groups and a resonance of the entire thorax that drives the wingbeat. Typical wingbeat frequencies are in the range from 100 Hz and even up to 1,000 Hz in the smallest species [5],[8].

**Figure 1:**
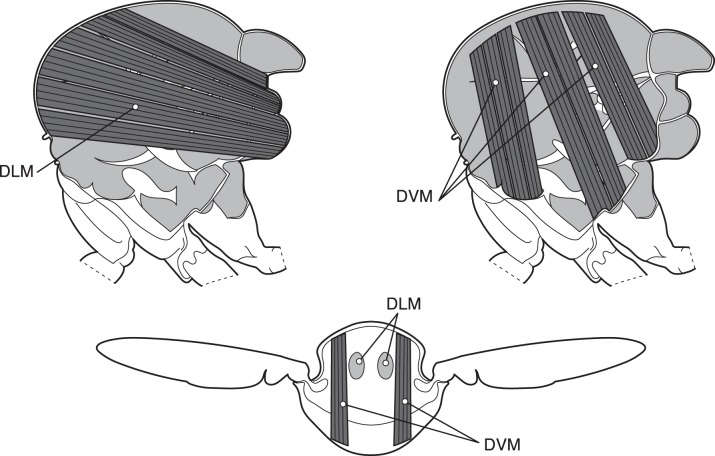
The thorax with and dorsal longitudinal (upper left) and dorso-ventral (upper right) power flight muscles of a fly. The cartoon (bottom) shows a transverse section through the thorax with dorso-ventral muscles (DVM) and dorsal longitudinal muscles (DLM) indicated. The two upper illustrations are redrawn from [6].

The forces from the flight muscles are transmitted to the wing through an intricate hinge mechanism (Figure 2). The hardened plates of cuticle between the thorax and wing (sclerites) are mobile and their positions relative to the thoracic outgrowths and wing determine the extent of the wing motion, i.e., the angular amplitude of the wingbeat [6].

**Figure 2:**
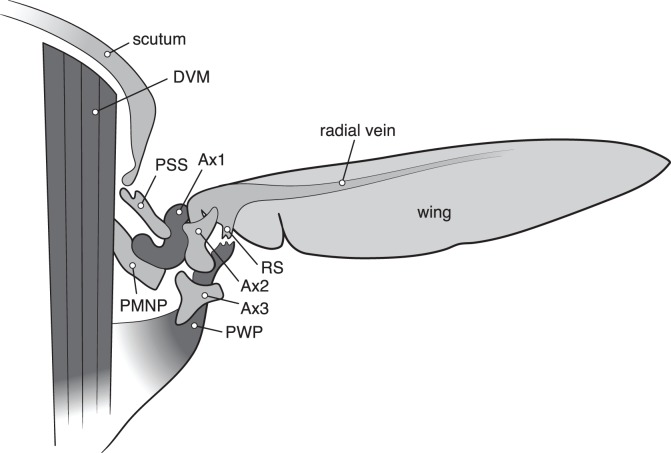
Cartoon illustration of a transverse section of the thorax of a fly in rear view, showing some elements of the complex wing hinge of a fly, consisting of ridges and protrusions on the thorax and a number of hardened plates of cuticle (sclerites) between the body (thorax) and the wing root. The basalare sclerite (not shown) is positioned anterior of the first axillary sclerite (Ax1). The indicated structures are dorso-ventral power muscle (DVM), pleural wing process (PWP), post-medial notal process (PMNP), parascutal shelf (PSS), axial wing sclerites (Ax1, Ax2, Ax3), and radial stop (RS). Redrawn and modified from [18].

Flight maneuvers arise owing to asymmetric force generation between the left and right wing. Aerodynamic force is proportional to the angle of attack (the angle between the wing surface and the airflow) and the speed squared relative to the air [9],[10]. Except from the turning points of each half-stroke, when the wings rotate about their span wise axes, the angle of attack is usually quite constant during the translational phases of the wingbeat [10], while asymmetric forces are mainly created by changing the wingbeat amplitude in flies [11]–[14]. With wingbeat frequency kept constant, changed amplitude changes the speed and hence force generated.

The control of the elements forming the hinge mechanism of the wing is achieved by the steering muscles, which are tiny in terms of mass (<3% of the power muscle mass), but mean everything when it comes to making flight maneuvers. In contrast to the power muscles the steering muscles are synchronous, i.e., there is a 1∶1 correspondence between neural spikes and muscle contraction. No less than some 22 pairs of steering muscles are involved in the force transmission; a few of these indirectly modulate the output by affecting the resonating properties of the thorax, while others are directly attached to the sclerite elements of the hinge mechanism [6],[15]. Three small muscles (b1–b3) are attached to the basalare plate that is directly involved in wing articulation (Figure 3). The actual wing sclerites (Figure 2) are also controlled by specific steering muscles, also with the function of moving the sclerites in relation to required wing motion. The main control function of the hinge mechanism appears to be of the downward movement of the wing, i.e., the angle at the turning point at end of downstroke. For a detailed review about the steering muscles and their function see Dickinson and Tu [6].

**Figure 3:**
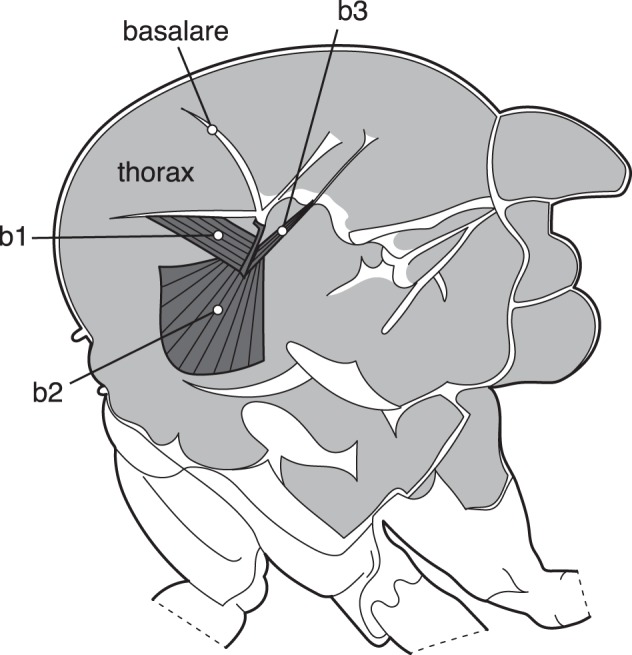
The position of the three steering flight muscles b1–b3 inserted to the nail-shaped basalare sclerite. Contraction by the b1 and b2 muscles move the basalare forward and their antagonist b3 moves it backwards when contracted. Redrawn from [6].

To date, the function of the steering muscles has been revealed mainly by electrophysiological studies on tethered subjects. Tethering means that the animal is glued to the end of a thin rod, often with force sensors attached to it, and then stimulated to “fly.” In many insects this can be achieved by simply blowing at them or placing them in a wind tunnel. On the tether the insect can either be presented with a visual stimulus or be rotated, which flies can sense via their halteres (hind wings modified to sensory gyroscopic sensory organs) [16]. By inserting electrode wires into the steering muscles, the neural impulses are measured at the same time as the wingbeat kinematics is recorded [13],[17]. What we know about the function of the steering muscles comes from the meticulous studies of correlations between muscle activity and the associated wing movement, including how the hinge mechanism works [6],[18]. Needless to say, such experiments are extremely difficult to achieve in small insects like blowflies and fruit flies that flap their wings at high frequencies. Recent studies of the wing and hinge kinematics provide some support for the hypothesis that the hinge may have a gear function that affects stroke amplitude, as well [18]. However, there are still many open questions regarding the exact function of the steering muscles and how they help in generating laterally asymmetric forces during a fly's flight maneuver [6].

In an article published in this issue of *PLOS Biology*, Walker and colleagues take a new approach for studying how steering muscles regulate the power output from power muscles [19], using time-resolved x-ray microtomography [20]. By rotating tethered blowflies (*Calliphora vicina*) in the X-ray beam, a 3D-movie was captured that shows how the steering muscles move. This by itself is a grand achievement at a wingbeat frequency of 145 Hz. As the flies could sense being rotated the steering muscles acted accordingly to achieve an asymmetric power output as a response to a perceived turn. The movies that accompany the article show how several of the key steering muscles and their sclerites operate in concert during the course of a wingbeat, and the visual results are supported by advanced statistical analyses of muscle strain rates and their phase offset. For example, the b1 and b3 muscles (Figure 3) work antagonistically, as was known before, but on the low-amplitude wing the oscillations are delayed by about a quarter of a wingbeat. The strain amplitudes of b1 and b3 were different between the two wings, which were found to be due to dorso-ventral movement of the basalare sclerite on the high-amplitude side and rotation on the low-amplitude side. This shows even higher complexity of the wing hinge than was previously envisaged.

The measurements of strain rate in the muscle confirmed the results of a previous study, which showed that asymmetric power output is partially achieved by negative work [21], i.e., absorption of work, by the b1 muscle on the low-amplitude wing. As with other muscles, the steering muscles insert on the skeletal parts and sclerites by tendons. The tendon of the muscle (I1) associated with the first axillary sclerite was observed to buckle when the wing was elevated above the wing hinge, indicative of compressive force acting on it near the top of the wing stroke. This buckling of the tendon forces a reinterpretation of the function of this muscle: it is involved in reducing stroke amplitude at the bottom of the downstroke rather than exerting stress near the opposite end of the stroke. Tendon buckling was seen in some other muscles as well, and although this is its first observation, it may be a more general mechanism involved in control of insect wingbeat kinematics.

What are the wider implications of this new study? First, it demonstrates the utility of a new approach to examine the *in vivo* operation of several insect flight muscles. This alone signals a methodological breakthrough that promises more. So far the flies were tethered and studied during one behavioral treatment (rotation about the yaw axis). Real flight maneuvers, however, also involve angular rotation about pitch and roll axes, acceleration, and braking. Thus, it remains to be seen how the steering muscles operate to control more subtle changes in wing kinematics during the turning saccades and advanced flight maneuvers that take place during free flight. The method involved exposure to lethal X-ray doses, which of course limits how long the experiments can be. Second, tethering is the prevailing paradigm for studying insect flight, but because it interrupts the sensory feedback loop [22], it would be useful for future studies to compare tethered and free flight in some commonly studied species. Furthermore, a more complete understanding of the flight muscle-hinge mechanism may help bio-inspired design of wing articulation systems for fly-like micro air vehicles. Until then, we can enjoy the stunning videos of the oscillating thorax and flight muscle system of the blowfly [19]. See the video from the related research article here (http://youtu.be/P6lBkK3J9wg) or [19].
